# Virtual treatment planning in three patients with univentricular physiology using computational fluid dynamics—Pitfalls and strategies

**DOI:** 10.3389/fcvm.2022.898701

**Published:** 2022-08-03

**Authors:** Marie Schafstedde, Pavlo Yevtushenko, Sarah Nordmeyer, Peter Kramer, Anastasia Schleiger, Natalia Solowjowa, Felix Berger, Joachim Photiadis, Yaroslav Mykychak, Mi-Young Cho, Stanislav Ovroutski, Titus Kuehne, Jan Brüning

**Affiliations:** ^1^Department of Congenital Heart Disease–Pediatric Cardiology, German Heart Center Berlin, Berlin, Germany; ^2^Institute for Cardiovascular Computer-Assisted Medicine, Charité–Universitätsmedizin Berlin, Berlin, Germany; ^3^Berlin Institute of Health, Berlin, Germany; ^4^German Centre for Cardiovascular Research, Partner Site Berlin, Berlin, Germany; ^5^Department of Cardiothoracic and Vascular Surgery, German Heart Center Berlin, Berlin, Germany; ^6^Department of Pediatric Cardiology, Charité–Universitätsmedizin Berlin, Berlin, Germany; ^7^Department of Congenital Heart Surgery, German Heart Center Berlin, Berlin, Germany

**Keywords:** Fontan, univentricular physiology, 4D flow MRI, computed tomography, image-based modeling, computational fluid dynamics, decision support, hepatic factor

## Abstract

**Background:**

Uneven hepatic venous blood flow distribution (HFD) to the pulmonary arteries is hypothesized to be responsible for the development of intrapulmonary arteriovenous malformations (PAVM) in patients with univentricular physiology. Thus, achieving uniform distribution of hepatic blood flow is considered favorable. However, no established method for the prediction of the post-interventional hemodynamics currently exists. Computational fluid dynamics (CFD) offers the possibility to quantify HFD in patient-specific anatomies before and after virtual treatment. In this study, we evaluated the potential benefit of CFD-assisted treatment planning.

**Materials and methods:**

Three patients with total cavopulmonary connection (TCPC) and PAVM underwent cardiovascular magnetic resonance imaging (CMR) and computed tomography imaging (CT). Based on this imaging data, the patient-specific anatomy was reconstructed. These patients were considered for surgery or catheter-based intervention aiming at hepatic blood flow re-routing. CFD simulations were then performed for the untreated state as well as for different surgical and interventional treatment options. These treatment options were applied as suggested by treating cardiologists and congenital heart surgeons with longstanding experience in interventional and surgical treatment of patients with univentricular physiology. HFD was quantified for all simulations to identify the most viable treatment decision regarding redistribution of hepatic blood flow.

**Results:**

For all three patients, the complex TCPC anatomy could be reconstructed. However, due to the presence of metallic stent implants, hybrid models generated from CT as well as CMR data were required. Numerical simulation of pre-interventional HFD agreed well with angiographic assessment and physiologic considerations. One treatment option resulting in improvement of HFD was identified for each patient. In one patient follow-up data after treatment was available. Here, the virtual treatment simulation and the CMR flow measurements differed by 15%.

**Conclusion:**

The combination of modern computational methods as well as imaging methods for assessment of patient-specific anatomy and flow might allow to optimize patient-specific therapy planning in patients with pronounced hepatic flow mismatch and PAVM. In this study, we demonstrate that these methods can also be applied in patients with complex univentricular physiology and extensive prior interventions. However, in those cases, hybrid approaches utilizing information of different image modalities may be required.

## Introduction

Initially introduced in 1971 as physiological repair for tricuspid atresia, the Fontan operation aims at abolishing cyanosis by entirely eliminating right to left shunt in patients with univentricular heart disease (1). Its contemporary modification, the staged total cavopulmonary connection (TCPC), has emerged as the standard procedure in the treatment of these patients (2, 3). However, persisting or reoccurring cyanosis is frequently observed (4) and the development of intrapulmonary capillary arteriovenous malformations (PAVM) is one of the possible underlying reasons. Particularly after superior cavopulmonary connection or Kawashima procedure, where hepatic venous flow is usually not directed into the pulmonary arteries, development of PAVM is reported in up to 25% of patients (5). Affected patients suffer from severe cyanosis and increased risk for morbidity and mortality (4–6).

Usually, regression of PAVM and clinical improvement is observed after completion of TCPC, when hepatic venous blood and thus the hypothesized “hepatic factor” is redirected into the pulmonary arteries again (7–9). In cases of incomplete regression or of development of unilateral PAVM after Fontan completion, an uneven distribution of hepatic venous blood flow (HFD) between both pulmonary arteries has been discussed to be responsible (10–15). This is supported by several case reports and studies that observed a resolution of PAVMs after surgical or interventional redirection of hepatic blood flow in Fontan patients (14, 16–18).

In patients with PAVM, the main challenge is to confidently provide a surgical or interventional strategy that will result in a more balanced HFD. Several treatment strategies in terms of various possible anatomical Fontan connections have yet been analyzed (19) and first approaches emerged, evaluating novel surgical treatment options for Fontan patients and envisaging patient-specific treatment planning using virtual manipulation of the patient-specific anatomy and subsequent calculation of hemodynamic results (20–22).

Computational fluid dynamics (CFD) offer the possibility to simulate different treatment strategies and perform outcome predictions, which might enhance and supplement the preoperative or pre-interventional interdisciplinary decision-making process. This is an evolving field with necessity for further validation, however, it might allow to optimize therapy planning and to achieve the desired hemodynamic result in a patient-specific case (23–25).

The heterogeneity of cardiovascular anatomies in Fontan patients may present a particular challenge to the surgical establishment of a Fontan circuit that results in an optimal HFD to both pulmonary arteries. This is particularly true for anatomies with interrupted inferior vena cava and vena azygos/hemiazygos continuation in which TCPC is usually completed by separately connecting the hepatic veins to the pulmonary arteries with an intracardiac tunnel or an extracardiac conduit (18, 26). Especially in these complex cases, virtual treatment simulations are of exceptional clinical interest.

The purpose of this study is hence to evaluate the potential benefit of virtual treatment planning in patients with univentricular heart malformations and PAVM development due to uneven hepatic flow distribution and to demonstrate feasible diagnostic algorithms using CFD simulations for individualized patient-specific surgical or catheter-based interventional therapy planning to optimize HFD as causal treatment rationale in these patients.

## Materials and methods

### Study design

Three patients with Fontan circulation and history of PAVM development were retrospectively analyzed. The presence of macroscopic PAVM was determined angiographically using criteria such as macroscopically identifiable pulmonary capillary bed and rapid pulmonary transit time of contrast agent. If technically possible, also pulmonary venous oxygen saturation was measured in the affected and unaffected lung. Due to severe cyanosis and limited physical capacity (objective and subjective), these patients were considered for catheter-based or surgical intervention to improve HFD. For all patients, cardiovascular magnetic resonance imaging (CMR) as well as computed tomography (CT) data was available. The study was approved by the institutional review board and the institutional ethics committee (decision number EA2/126/15). Individual informed consent was waived.

### Cardiovascular magnetic resonance imaging acquisition

Cardiovascular magnetic resonance imaging imaging was performed using a 1.5 Tesla CMR system (Achieva; Philips Medical Systems, Best, Netherlands) with a 5-element cardiac phased-array coil. Balanced 3D steady-state-free-precession (SSFP) imaging during end-diastole (3 signal averages, navigator gated, ECG triggered) and cine-sequences were used for reconstruction of the patient-specific anatomy. Flow volume quantification was performed using 2D free-breathing phase contrast cine CMR sequences of all vessels up- and downstream of the Fontan circulation, i.e., the inferior vena cava (IVC), superior vena cava (SVC), right pulmonary artery (RPA), left pulmonary artery (LPA), azygos/hemiazygos vein, innominate vein as appropriate, as well as the aorta. Acquired and reconstructed voxel resolutions of these measurements were 2.3 × 3.1 × 7 mm^3^ and 1.1–2.2 × 1.1–2.2 × 7 mm^3^, respectively. Repetition time, echo time, flip angle were 5.1–5.4 ms, 3.0–3.3 ms, 15°, respectively. The velocity encoding of venous vessels was 100 cm/s, whereas 200–400 cm/s were chosen for arterial vessels as appropriate. Aliasing was ruled out in all measurements. All flow measurements were completed with automatic correction of concomitant phase errors.

### Computed tomography imaging acquisition

All CT images were acquired using the same dual-source multi-slice spiral computed tomography (CT) scanner (Somatom Definition Flash, Siemens Healthcare GmbH, Erlangen, Germany). Tube currents were adapted individually, whereas a constant tube voltage of 100 kV was used. Spatial resolution of the CT images varied from 0.48 × 0.48 mm^2^ to 0.62 × 0.62 mm^2^, whereas the slice thickness was 0.7 mm for all three patients.

### Image post-processing and generation of pre-interventional anatomical models

The patient-specific, pre-interventional anatomy was reconstructed using ZIBAmira (v. 2015.28; Zuse Institute Berlin, Germany). Here, the CT images were used preferably due to better contrast, resolution, and lesser influence of metallic artifacts caused by implants such as stents. However, the inflow from the SVC, and thus the contrast agent distribution, was often uneven. Whereas some regions were flooded by contrast agent, resulting in artifacts due to strong contrast gradients, other regions were not perfused, resulting in poor contrast between vessels and surrounding tissue. Here, SSFP CMR images were used to reconstruct areas that were insufficiently distinguishable in the CT data.

**FIGURE 1:**
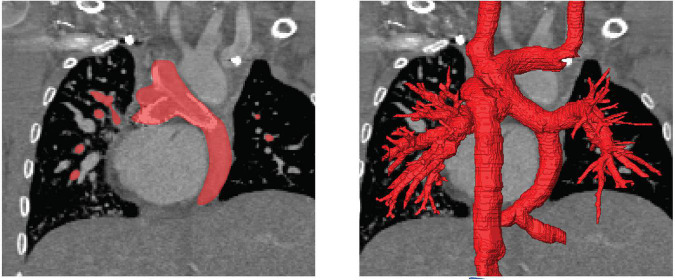
Illustration of the image-reconstruction procedure. Image voxels belonging to the region of interest were selected either manually or using semi-automatic approaches as for example region growing algorithms based on image intensity as well as local intensity gradients **(left)**. This procedure was performed for all slices and all three reconstruction orientations, to obtain the three-dimensional label mask of the patient-specific anatomy **(right)**.

The same methods for image processing and subsequent reconstruction of the patient-specific Fontan circulation were used for MR as well as CT images. Due to the large heterogeneity of the image data, artifacts from metallic stents and contrast agent, regions of interest were selected mostly manually, however, semiautomatic methods such as threshold-based selection of connected regions were used whenever possible. However, no fixed Hounsfield or grayscale threshold was used, but the threshold had to be adapted to the respective image region due to changes in contrast. In general, Hounsfield units between 150 and 200 were used as threshold, in well-contrasted regions of the CT data. Image data was reconstructed by slicing through all three orientations repeatedly. First, the veins and grafts leading into the Fontan circulation, i.e., the SVC and IVC as well as the azygos/hemiazygos vein were reconstructed, followed by the pulmonary arteries. For the validation of reconstructions, angiographic images were additionally evaluated, as this image modality allows for accurate measurement of diameters as well as visualization of anastomosis sites (Figure 1).

Anatomical reconstructions from CT and CMR data were registered using the vessels that were identifiable within both image modalities, which were usually the SVC and IVC. Then, a joint reconstruction of both image modalities was generated by super-imposition. Subsequently, a surface geometry was generated from this reconstruction using a marching cubes algorithm (27). This surface geometry was smoothed using a volume preserving smoothing algorithm (28). Smaller topological errors, as for example non-manifold vertices that resulted from vessels being close to each other, were corrected using Meshmixer (v. 3.5; Autodesk, Mill Valley, CA, United States). This tool was also used to truncate all vessel endings perpendicularly.

Reconstruction of the patient-specific anatomy of the TCPC was conducted by two engineering researchers with several years of experience in image-based reconstruction. They were supported by surgeons and cardiologists by joint evaluation of information available from previous surgical and other reports to better understand the complex anatomy of the TCPC, respective vessels and to select suitable imaging sequences for reconstruction.

### Generating surgical and interventional options

For each case, different surgical, catheter-based interventional, and in some cases combined treatment strategies had been proposed after discussion in the institutional heart team that includes interventional and non-interventional cardiologists, as well as congenital cardiac surgeons. These strategies were then virtually implemented by manipulation of the pre-interventional anatomy. While these virtual manipulations were performed by engineering researchers experienced in cardiovascular simulations, all virtual interventions were performed in attendance of at least one heart team member and finally approved by the whole heart team.

The treatment options varied from implantation of covered and bare metal stents, implantation of vascular grafts, resection of existing vascular connections with re-anastomosis at an altered position as well as vascular patch augmentation or reconstruction. Treatment strategies were performed if they were considered feasible. Assessment of feasibility was mainly based on the experience of the heart team member in favor of the respective strategy and subsequent discussions of the entire heart team. In addition to that, several procedural aspects were evaluated: for vascular grafts a pathway not intersecting any relevant structures had to exist; for stent implantation, vessel diameters had to have the right size allowing anchoring of the stent; for vascular graft implantation, the anastomosis site had to be free from scar tissue and not located to close to a stent. All treatment options were only performed in a qualitative manner, no constitutive models were used to describe anatomical properties.

**FIGURE 2:**
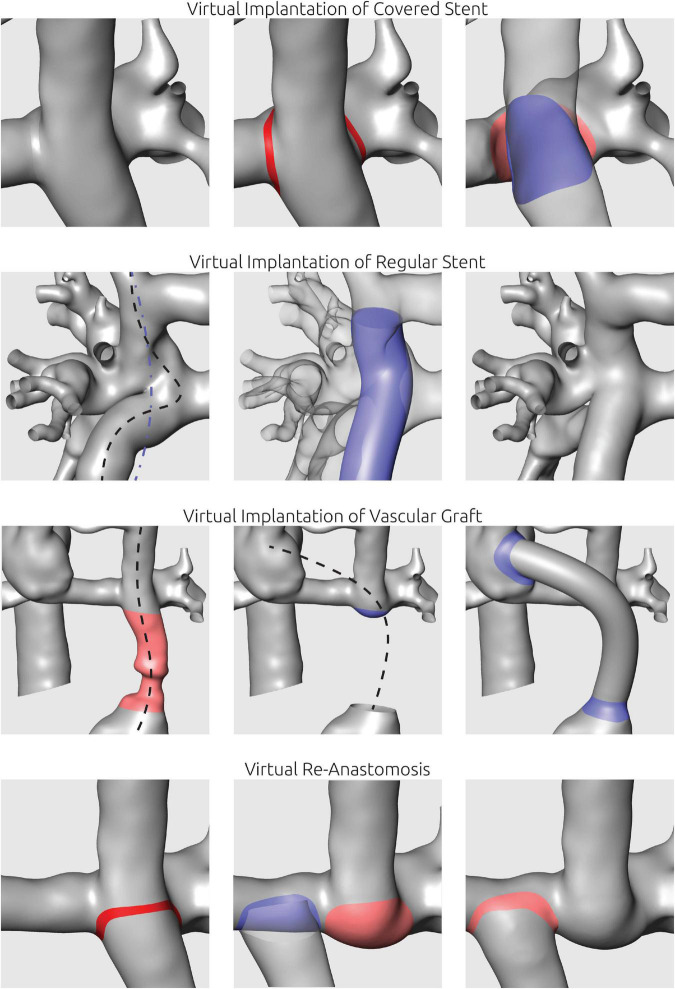
Illustration of virtual manipulations to mimic interventional or surgical procedures. Virtual implantation of covered stents was utilized to separate two vessel lumina. Virtual stent implantation aimed at straightening of curved vascular paths using a manually defined spline (blue line). To mimic surgical implantation of vascular grafts, the respective vessel section was replaced using a tube following a manually positioned centerline. For virtual re-anastomosis, vessels were separated and shifted to the new anastomosis site.

To facilitate these virtual treatments, all manipulations were performed using Meshmixer. Here, resection of vessels was performed by selection and removing of surface elements. Similarly, the implantation of a covered stent to redirect blood flow was performed by separation of the connection between vessels to be bridged and subsequent closure of these vessels to facilitate two separate lumina. Vascular grafts were first designed in ZIBAmira using a function to generate and manipulate cubic splines in 3D by movement of the different points of the spline. Thus, the path of the vascular graft could be specified considering the surrounding tissues by superimposition of the CT image information. After the path was specified, a tube with a given diameter was generated following this path. Implantation of the graft to the pre-interventional anatomy was then performed using Meshmixer. For implantation of bare metal stents aiming at straightening of vessel paths and enlarging the vessel cross sectional area, the center line of the stent was determined similarly to the vascular graft implantation. Then, a tube with a given length and diameter was generated following this center line. This tube was used as attractor and the vessel surface was deformed to match the stent orientation. Examples of these different procedures are shown in Figure 2.

### Numerical simulation of hepatic blood flow distribution

Simulation of patient-specific blood flow was performed using the finite volume CFD solver STAR-CCM + (v15.4, Siemens PLM, Plano, TX, United States). The pre-interventional as well as virtually treated surface geometries were imported, and all truncated vessel openings were closed for subsequent definition of boundary conditions. At all vessels except the pulmonary arteries, a mass flow boundary condition was specified. Here, the average mass flow rate measured from the 2D VEC CMR was specified. For the LPA and RPA, outflow boundary conditions were applied, which allow the specification of the percentage of mass flow passing through these vessels. The ratio between LPA and RPA was calculated from the volume flow rate measurements in the respective vessels if available.

The vessel walls were assumed to be rigid, and a no-slip boundary condition was applied. Blood was modeled as non-Newtonian fluid with a shear-dependent viscosity described using a generalized Carreau-Yasuda model (29, 30) and a constant density of 1050 kg/m^3^.

A polyhedral mesh with a base size of 0.6 mm and a boundary layer consisting of four adjacent prism layers was generated. The overall thickness of the boundary layer was 30 percent of the base size with each prism layer’s height being 30 percent larger than the previous one. No specific refinement regions were specified, but the meshing algorithm was able to automatically reduce the base size, as for example in regions featuring high curvatures.

Simulations were considered converged when static pressure values at the pulmonary artery outlets were stable and residuals of momentum and continuity were below 1e-5.

**FIGURE 3:**
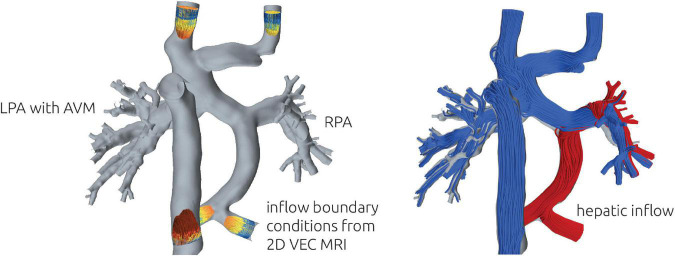
Illustration of the numerical setup (left). At all vessels except LPA and RPA, the patient-specific volume flow rate measured using 2D VEC CMR was specified as boundary conditions. 2D VEC CMR measurements at the LPA and RPA were used to specify the relative ratio of blood leaving the domain through each respective vessel. To illustrate the distribution of hepatic blood flow, streamlines of different color were seeded from the respective vessels (right). The example illustrates a total separation, with no hepatic blood (red) reaching the LPA.

To calculate HFD, massless particles were seeded in the hepatic vein once the simulation was converged. Then, the simulation was continued until a stationary distribution of particles leaving the domain through LPA and RPA was achieved. In this study, HFD will be reported rounded to multiples of 5 percent (Figure 3).

## Results

### Patient characteristics

Patients’ characteristics at baseline are depicted in Table 1. Due to an interrupted IVC, in two of three patients a Kawashima procedure had been performed. Right ventricular morphology of the systemic ventricle was present in two patients. Oxygen saturation levels were 81–83% at rest. Physical capacity was reduced in all patients according to cardiopulmonary exercise testing and New York Heart Association (NYHA) functional class ranged from II to III.

**TABLE 1:** Clinical summary of all patients.

	Patient #1	Patient #2	Patient #3
**Patient characteristics at Fontan operation**			
Age (years)	3.4	4.2	2.4
Sex	Female	Male	Male
**Anatomical data**			
Cardiac morphology	Complex DORV with ventricular imbalance	ubAVSD, DORV, CoA, LSVC, dextrocardia, interruption of the inferior vena cava with right-sided azygos continuity	ubAVSD, d-TGA, PS, LSVC, laevocardia, interruption of the inferior vena cava with left-sided hemiazygos continuity
Morphology of systemic ventricle	LV	RV	RV
Heterotaxy	No	Yes Left atrial isomerism, polysplenia	Yes Left atrial isomerism, polysplenia, situs inversus abdominalis
**Surgical data**			
Type of Fontan completion	Superior cavopulmonary anastomosis, extracardiac TCPC	Bilateral superior cavopulmonary anastomosis (right-sided Kawashima and bidirectional Glenn anastomosis, extracardiac hepatic vein conduit left-sided)	Bilateral superior cavopulmonary anastomosis (left-sided Kawashima and bidirectional Glenn anastomosis, extracardiac hepatic vein conduit right-sided)
**Follow-up data**			
FU-duration (years)	19.8	14.2	9.8
Age at last follow-up (years)	23.2	18.4	12.2
Weight at last follow-up (kg)	58	68	44
Height at last follow-up (cm)	163	176	148
BSA at last follow-up	1.6	1.8	1.4
NYHA I-IV at last follow-up	II	III	III
Hemoglobin at last follow-up (g/dl)	17.5	20.1	15.2
Oxygen saturation at rest	81	83	80
**Hemodynamic data**			
PAP (mmHg)	13	12.5	10
Site of PAVM	Right upper lobe	Right lung	Left lung
Residual shunts	PAVM	small venovenous collaterals, PAVM	PAVM
Implanted prosthetic material	Coils, Amplatzer septal occluder, CoA Stent	CoA Stent, peripheral stent graft (hepatic vein conduit), LPA-Stent	Coils, Amplatzer septal occluder, CoA Stent, RPA-Stent, LPA Stent

BSA, body surface area; CoA, aortic coarctation; DORV, double outlet right ventricle; d-TGA, right sided transposition of the great arteries; LSVC, left persistent superior vena cava; LV, left ventricle; NYHA, New York Heart Association; PAVM, intrapulmonary arteriovenous malformations; PS, pulmonary valve stenosis; RER, respiratory exchange ratio; RV, right ventricle; TCPC, total cavopulmonary connection; ubAVSD, unbalanced atrioventricular septal defect; VE/VCO_2_ slope, slope of ventilation/carbon dioxide production ratio; VO_2_ peak, peak oxygen uptake.

In one patient, re-routing surgery has already been performed meanwhile. Clinical follow-up examinations for this patient were performed 1 and 6 months after surgery and revealed good results in terms of improved physical capacity (now NYHA I) and oxygen saturation levels (currently 90% at rest).

### Patient #1–Anatomy and hemodynamic considerations

This patient has a complex transposition of the great arteries with double outlet right ventricle, right ventricular hypoplasia and coarctation of the aorta. The medical history includes a banding of the pulmonary artery, an aortic arch patch augmentation, the creation of a bidirectional Glenn anastomosis and finally an extracardiac TCPC with fenestration, which has been closed by catheter intervention during follow-up. Due to significantly decline of oxygen saturation, invasive diagnostic assessment was performed revealing that the Fontan conduit with the hepatic venous flow is directed mainly to the left side of the lung and the right lower lobe, whereas the upper and middle right pulmonary lobe is supplied by the SVC alone (see Figure 5, top row). Consecutively, relevant macroscopically identifiable PAVM developed in the right upper and middle pulmonary lobe.

### Patient #1–Image-based reconstruction of the patient-specific circulation

**FIGURE 4:**
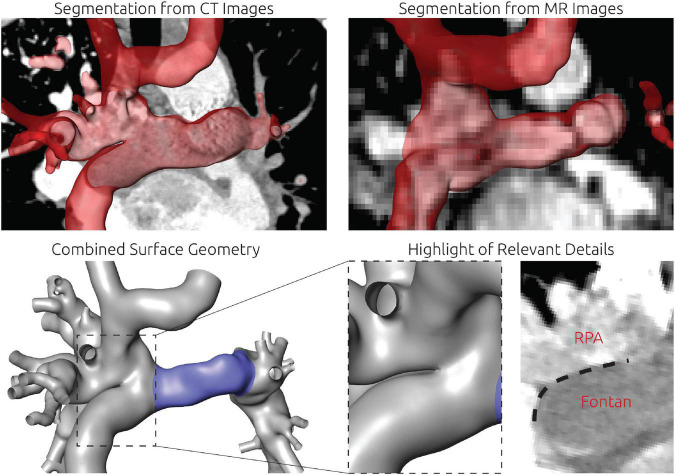
Detailed overview of the segmentation strategy for the first patient. The upper left and right panel show the segmentation from CT and CMR images, respectively. The lower left panel shows the final surface geometry that was reconstructed by combination of both segmentations. Tissue highlighted in blue was reconstructed from CMR images, whereas the remaining tissue was reconstructed from CT images. The lower right panel highlights the complex connection between RPA, SVC, and Fontan conduit, which could only be discriminated in CT images due to the distinct Hounsfield gradient from Fontan conduit to RPA.

For this patient, reconstruction of the patient-specific circulation was performed using CT and CMR images (see Figure 4). In general, CT images allowed reconstruction of all relevant vessels but the proximal part of the LPA. Similar to the findings during angiography, the contrast agent administered during CT acquisition mainly reached the right upper and middle pulmonary lobe. Due to the lack of contrast agent in the proximal LPA as well as metallic artifacts of the aortic stent, neither the cross-section nor the orientation of the vessel could be discerned with confidence. Here, CMR images were used to compensate the lack of information. The complex anatomy of the anastomosis between the Fontan conduit and the RPA could only be discerned from CT images using the Hounsfield unit gradient as orientation (see Figure 4, bottom right). Resolution of the CMR images was not sufficient to determine this anatomical detail.

**FIGURE 5:**
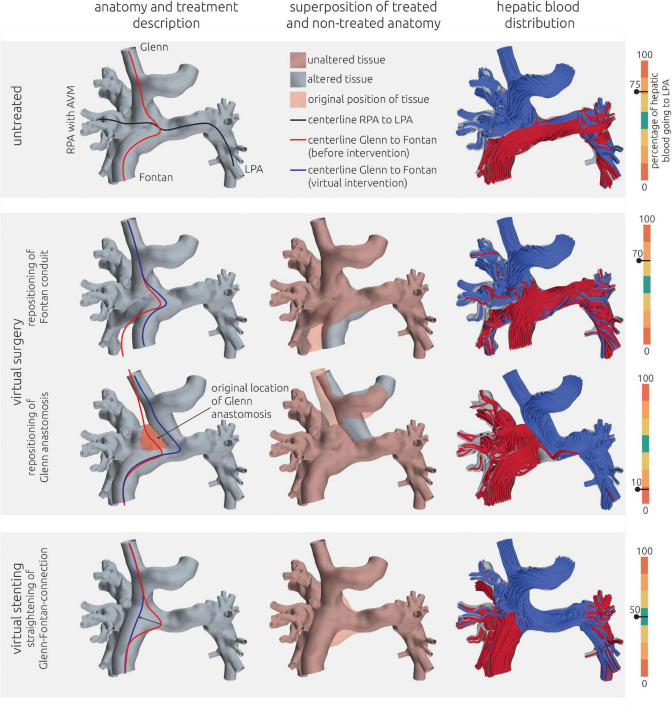
Visualization of the estimated distribution of hepatic blood flow to the pulmonary arteries for the untreated patient-specific anatomy (top row) as well as two surgical (middle rows) and one interventional treatment option (bottom row) for patient 1. In the left column, the anatomy of the conduit is shown together with centerlines from LPA to RPA (black line) and from SVC to IVC. The original path of the connection from SVC to IVC is highlighted in red, whereas the altered path after virtual treatments is shown in blue. In the central row, the untreated anatomy is superimposed as transparent red surface over the virtually modified anatomy, highlighting all regions that were altered. The right column shows the calculated distribution of hepatic blood (red) into the LPA and RPA, as well as the distribution of blood coming from other veins (blue). Hepatic blood flow distribution is indicated by the gauge to the right.

### Patient #1–Virtual treatment and hemodynamic simulations

The pre-interventional simulations match the clinical evaluations: 75% of the hepatic blood flow reaches the LPA, with the remaining fraction also perfusing the right lower pulmonary lobe. No perfusion of hepatic blood in the right upper and middle pulmonary lobe was observed. The complex shape of the anastomosis between Fontan conduit and RPA seems to divert the blood from the IVC and the hepatic veins directly toward the LPA, while the blood flow from the SVC is oriented perpendicularly onto the wall of the Fontan conduit and directed toward the right upper and middle lobe.

For this patient, two surgical and one interventional treatment options were considered feasible. All options aimed at mitigating the effects of the complex anastomosis between Fontan conduit, the superior cavopulmonary anastomosis and RPA. The virtual changes in the patient-specific anatomy as well as the hemodynamic results in terms of streamlines and HFD mismatch are shown in Figure 5 for all options.

As the first virtual surgical treatment option a translocation of the hepatic vein conduit toward the LPA was performed. Here, the main focus was to alter the angle and the bend of the anastomosis and to remove the flap-like structure between Fontan conduit and RPA. However, this intervention did not result in any relevant improvement of HFD mismatch, with 70% of the hepatic blood flowing to the LPA and the majority of the remaining hepatic blood still only reaching the right lower pulmonary lobe.

The second surgical option aimed at redirecting the SVC blood flow toward the LPA by re-anastomosis of the SVC to a position closer to the LPA. Thus, the SVC blood would not impinge on the wall of the Fontan conduit. This method resulted in an almost complete reversal of HFD, meaning that the LPA was now mainly perfused by the SVC, whereas the hepatic blood went almost entirely to the RPA, with only 10% reaching the LPA.

The interventional option aimed at straightening the connection from SVC to the Fontan conduit using a stent. Here, the idea was to anchor a stent in both vessels and to stretch the flap-like structure, which orients the hepatic blood flow toward the LPA, to construct a more perpendicular intersection between the vessels. This intervention resulted in an even HFD toward LPA and RPA.

### Patient #2–Anatomy and hemodynamic considerations

The second patient has an unbalanced atrioventricular septal defect, double outlet right ventricle, coarctation of the aorta, left persistent superior vena cava (LSVC), dextrocardia and interruption of the IVC with right-sided azygos continuity. Additionally, the patient has heterotaxy with left atrial isomerism and polysplenia. This complex anatomy was ultimately palliated by creating a bilateral superior cavopulmonary anastomosis (right-sided Kawashima and left-sided bi-directional Glenn anastomosis) with an extracardiac, left-sided hepatic vein conduit. Initially a fenestration was created, that was closed again several months later.

During follow-up, reduced physical capacity and declining oxygen saturation levels were observed and an invasive diagnostic assessment was performed. Next to a pronounced aneurysm that has formed at the junction of the azygos vein with the SVC, a significant stenosis in the central pulmonary artery between the RPA and LPA could be detected. Due to this fact, there was an almost exclusive flow from the left-sided hepatic vein conduit toward the LPA, whereas the RPA was only supplied by the right-sided Kawashima anastomosis (see Figure 7 top row). Here, angiography revealed significant PAVM with oxygen saturation levels as low as 70% in the right pulmonary veins. However, to the implantation of a stent into the central pulmonary artery did not result in the redirection of hepatic blood toward the right side of the lung. Transcutaneous oxygen saturation levels are 83% at rest and decrease to 63% during physical exercise.

### Patient #2–Image-based reconstruction of the patient-specific circulation

**FIGURE 6:**
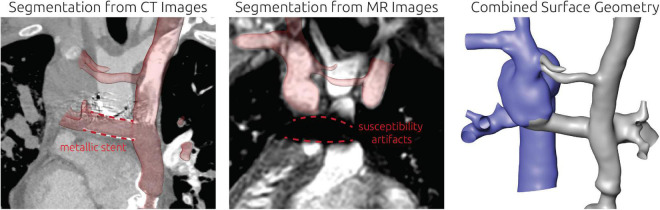
Detailed overview of the segmentation strategy for the second patient. The left and mid panel show the segmentation from CT and CMR images respectively. The right panel shows the final surface geometry that was reconstructed by combination of both segmentations. Tissue highlighted in blue was reconstructed from CMR images, whereas the remaining tissue was reconstructed from CT images. Additionally, the location of a metallic stent in the TCPC is highlighted in the CT images together with the resulting magnetic susceptibility artifacts during CMR imaging.

Similar to patient #1, image data from CT and CMR was required to reconstruct the entire TCPC. The reconstruction approach is shown in Figure 6. During CT, the inflow of the contrast agent *via* the Glenn anastomosis did not reach the right part of the circulation, resulting in very poor contrast of the azygos vein, the SVC as well as the RPA. Especially the aneurysm which formed at the junction from SVC, LPA and azygos was not discernable. In contrast, the connection between the left and right side of the circulation was not discriminable within the CMR images due to susceptibility artifacts caused by the stent in the communicating area between LPA and RPA. However, contrast in the CMR images allowed reconstruction of the right side of the circulation.

**FIGURE 7:**
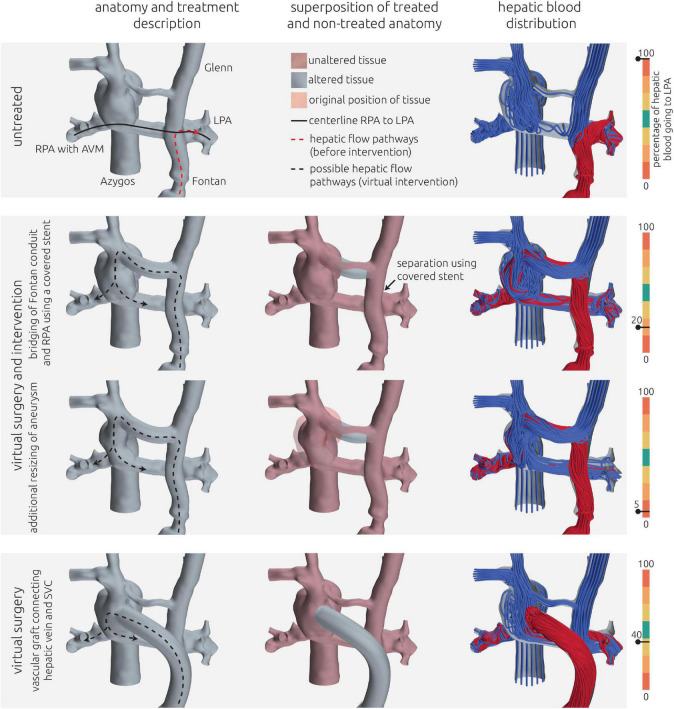
Visualization of the estimated distribution of hepatic venous blood flow to the pulmonary arteries for the untreated patient-specific anatomy (top row) as well as two combined approaches using surgery and interventional techniques (middle rows) and one surgical treatment option (bottom row) for patient 2. In the left column, the anatomy of the conduit is shown together with the centerline from LPA and RPA (constant line) and the possible flow pathways for hepatic blood (dashed lines). The original pathways are highlighted in red, whereas the altered pathways after virtual treatment are shown in black. In the central row, the untreated anatomy is superimposed as transparent red surface over the virtually modified anatomy, highlighting all regions that were altered. The right column shows the calculated distribution of hepatic blood (red) into the LPA and RPA, as well as the distribution of blood coming from all other veins (blue). Hepatic blood flow distribution is indicated by the gauge to the right.

### Patient #2–Virtual treatment and hemodynamic simulations

For this patient, two options for a combined surgical and interventional approach as well as one purely surgical approach were discussed. All approaches aimed at redirection of the hepatic blood flow toward the RSVC (right superior vena cava) and RPA. These treatment options as well as their predicted hemodynamic results are shown in Figure 7.

The pre-interventional simulation agreed well with the angiographic assessment of HFD: The entirety of the inflow from the hepatic vein conduit flowed to the LPA. The LPA was also perfused by the majority of the blood deriving from the LSVC. Similarly, the majority of the blood flow from the azygos vein and the RSVC flowed toward the RPA.

Both approaches using surgical as well as interventional techniques were similar in the main approach: using a covered stent, the hepatic vein conduit could be separated from the LPA, resulting in a direct connection between LSVC and the hepatic vein conduit. As the innominate vein connecting LSVC and RSVC in this patient is very hypoplastic, its diameter was considered insufficient to conduct the blood flow coming from LSVC and the hepatic vein conduit toward the RSVC. Thus, the innominate vein was intended to be replaced using a vascular graft with a diameter of 16 mm.

Additionally, a reduction of the aneurysm at the junction of azygos vein, RSVC and RPA was discussed, subsequently modeled, and simulated as second treatment option. The possible flow directions of blood coming from the hepatic vein conduit are depicted in Figure 7 compared to the original blood flow directions to illustrate the intended redirection of blood flow.

Both methods resulted in similar virtual post-interventional hemodynamics. The HFD was almost entirely reversed, meaning that most of the blood from the hepatic vein conduit was directed toward to the RPA. Resizing of the aneurysm further enhanced this effect from 20% of the hepatic blood reaching the LPA to only 5%.

Additionally, an entire surgical approach was discussed. Here the Fontan conduit was to be disconnected from the LPA, whereas the connection between LSVC and LPA remained. Then, a vascular graft with a diameter of 20 mm was intended to be used to connect the hepatic veins directly with the aneurysm at the Kawashima anastomosis. The path of this vascular graft was determined using the volumetric CT information to ensure that neither structures of the heart nor the lungs were compressed. Even though inflow from the LSVC still was directed mainly to the LPA, this strategy resulted in a sufficiently even HFD of 40%.

### Patient #3–Anatomy and hemodynamic considerations

The third patient has an unbalanced atrioventricular septal defect with dominant right ventricle, d-transposition of the great arteries, pulmonary stenosis, LSVC, and interruption of the IVC with left-sided hemiazygos continuity, heterotaxy with left atrial isomerism, polysplenia and situs inversus abdominalis. This complex anatomy was surgically palliated by creating a bilateral superior cavopulmonary anastomosis (left-sided Kawashima and bidirectional Glenn anastomosis) with an extracardiac right-sided hepatic vein conduit. Several cardiac catheterizations with embolization of hemodynamic relevant venovenous collaterals became necessary during follow-up. Additionally, multiple interventions with balloon dilatations and ultimately stent implantations in the RPA and LPA, due to stenoses in both vessels had to be performed. CMR-based flow measurements and angiography showed that the hepatic blood is mainly directed toward the RPA, whereas blood from the azygos vein, which is connected to the SVC shortly above its junction with the LPA, and blood from the upper half of the body is mainly directed to the LPA (see Figure 9 top row). Consecutively, relevant left-sided PAVM developed. Transcutaneous oxygen saturation values are approximately 80% at rest and decreased to 72% during physical exercise.

### Patient #3–Image-based reconstruction of the patient-specific circulation

**FIGURE 8:**
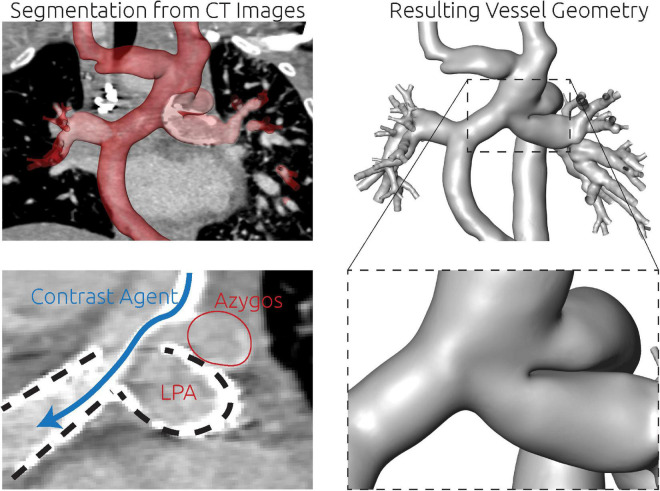
Detailed overview of the segmentation strategy for the third patient. The whole anatomy was reconstructed from CT images (top row). There were two metallic stents in the LPA and RPA (bottom left, dashed lines). Furthermore, the contrast agent deriving from the SVC was unevenly distributed and visible as a high contrasted band flowing toward the RPA (blue arrow). Directly superior to this complex region, also the azygos vein connected to the SVC. The separation between LPA and azygos vein could only be discriminated by the location and orientation of the LPA stent.

For this patient, the entire patient-specific anatomy was reconstructed using CT image data. As metallic stents were present in both LPA and RPA, the majority of the circulation of interest was not discriminated in CMR images due to susceptibility artifacts. While these stents also caused artifacts in the CT images, their contours were perfectly visible, allowing accurate reconstruction of the intricate connection between azygos vein, Glenn, and LPA, which is highlighted in the bottom right panel of Figure 8.

**FIGURE 9:**
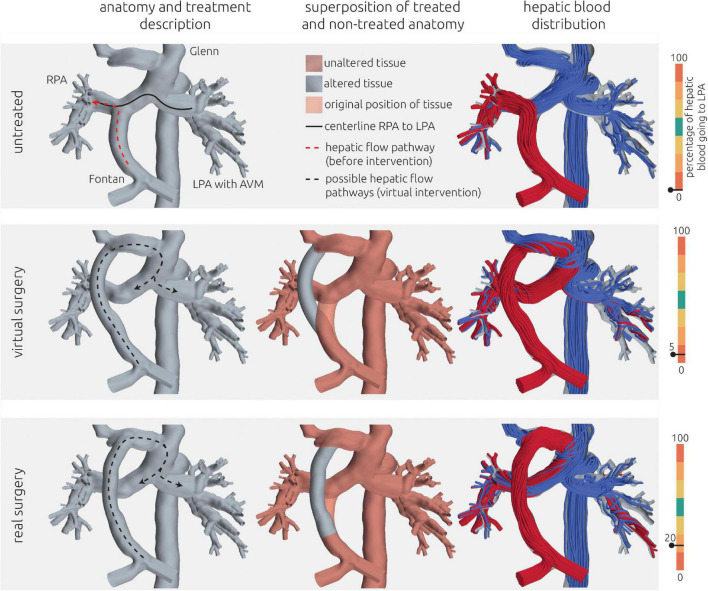
Visualization of the estimated distribution of hepatic venous blood flow to the pulmonary arteries for the untreated patient-specific anatomy (top row), a virtual surgical approach (middle row) as well as the real surgical result (bottom row) for patient 3. In the left column, the anatomy of the conduit is shown together with the centerline from LPA to RPA (constant line) and the possible flow pathways for hepatic blood (dashed lines). The original pathways are highlighted in red, whereas the altered pathways after treatments are shown in black. In the central row, the untreated anatomy is superimposed as transparent red surface over the virtually modified anatomy, highlighting all regions that were altered. The right column shows the calculated distribution of hepatic blood (red) into LPA and RPA, as well as the distribution of blood coming from all other veins (blue). Hepatic blood flow distribution is indicated by the gauge to the right.

### Patient #3–Virtual treatment and hemodynamic simulations

Also in this case, the pre-interventional simulation confirmed angiographic information obtained earlier (see Figure 9, top panel) with regard to distribution of hepatic blood.

Since the patient suffered from pronounced cyanosis and severely limited exercise capacity (NYHA functional class III) with signs of progressive heart failure, the indication for re-rerouting surgery was made. Due to the anatomical conditions, only one treatment option was considered feasible by the interdisciplinary Heart Team. This option was to re-route the hepatic vein conduit to the innominate vein (see Figure 9 middle and bottom row). The aim of this treatment option was to join all incoming blood flows and allow mixing of the hepatic blood before entering the pulmonary vasculature. While an improvement in HFD could be achieved in the virtual surgery planning, still only 5% of the hepatic blood was predicted to flow toward the LPA by the simulation.

However, in lack of alternative options, this surgical intervention was performed nonetheless on the patient and a 1-month follow-up including CMR assessment was performed. Using this CMR information, a comparison of the postoperative result with the preoperative model was performed. The conduit graft that was implanted to connect the hepatic veins with the innominate vein was reconstructed and merged with the pre-interventional reconstruction to obtain a digital representation of the real intervention. Minor differences in the orientation of the vascular graft as well as its anastomosis site to the innominate vein were observed compared to the virtual treatment. Additionally, 2D VEC CMR measurements were available from the follow-up measurements and were used as boundary conditions for simulation of the real surgical outcome. While the anatomy is very similar between real and virtual surgery, the real surgery resulted in an improved HFD with 20% reaching the LPA. The calculated hemodynamics of the virtual intervention and the real intervention using the follow-up information are shown in Figure 9.

## Discussion

In this study, methods for virtual treatment planning for interventional or surgical correction of Fontan patients were evaluated using routine data available for three patients suffering from severe cyanosis due to pronounced PAVM. All patients had a complex treatment history, resulting in multiple metallic implants in the Fontan circulation as well as neighboring vessels. Due to the resulting metallic artifacts as well as the complex anatomy of anastomosis sites, CT data was required to reconstruct the patient-specific anatomy, as it offered well-resolved image information for accurate reconstruction. However, in two cases additional information from CMR images was required to adequately reconstruct the complete Fontan circulation, as CT contrast was poor in some regions due to the uneven distribution of contrast agent as well as artifacts caused by metallic stents.

Simulation of pre-interventional hemodynamics confirmed angiographic assessment in all three cases and revealed hepatic blood flowing entirely to the pulmonary arteries without PAVM. These findings are consistent with the proposed mechanism between HFD and development of PAVM and further support previous studies, suggesting that numerical assessment of pre-interventional hemodynamics in Fontan circulation is feasible (20).

During interdisciplinary Heart Team meetings, at least one surgical, interventional, or combined treatment option was identified that was considered technically feasible and resulted in an improvement of HFD by virtual intervention. However, also treatment options with undesirable outcome, as for example entire reversal of HFD in patient #1 and #2, where the hepatic blood would flow almost exclusively toward the pulmonary arteries with PAVM, were identified. While even in those cases, a fraction of hepatic blood would reach the other pulmonary arteries, these findings highlight the advantages of pre-interventional virtual therapy planning as outcomes cannot be predicted intuitively. In the third patient, the only viable treatment option resulted only in moderate redirection of hepatic blood flow in the virtual and real surgery respectively. Even though the HFD was still predominantly biased toward the LPA, improvement of oxygen saturation could be observed in both follow-up examinations 1 and 6 months postoperatively. This suggests that already small improvements of HFD can be sufficient for regression of PAVM.

### Implications for the reliability of surgical planning

To date, there is promising but limited data on experience of virtual Fontan surgical planning. In its current state, virtual surgical planning was demonstrated to assess preoperative hemodynamic conditions, to identify anatomical constraints for potential surgical options, and to produce descent postoperative predictions if boundary conditions are similar enough between pre- and postoperative anatomy (20, 22, 31–33).

While Sundareswaran et al. were the first describing the use of surgical planning to correct PAVM in 2009, their work focusses on a single case report without postoperative imaging data to validate their simulated predictions (34). In 2012, Haggerty et al. successfully compared HFD between the predicted and post-operative states for four patients (25). Trusty et al. finally conducted the first prospective Fontan surgical follow-up study analyzing the accuracy of surgical planning based on 12 Fontan patients in 2019 (21). They published one of the largest patient cohorts and have many years of experience in this field of research, however, they also emphasize the need for sufficient imaging quality as one of the most essential prerequisites for accurate treatment planning. In this context it is the purpose of our work to demonstrate methodological approaches to also integrate complex cases with artifact-rich imaging as exactly these patients might benefit the most from interdisciplinary virtual therapy planning approaches.

There certainly is the need for further follow-up and thorough validation studies to encourage the translation of virtual therapy planning into the clinical routine. However, our data demonstrates that virtual therapy planning might have the potential to be a useful tool for single ventricle virtual therapy planning with the ability to deliver clinically relevant patient individual treatment options.

A relevant next step is the evaluation of how accurately a virtually planned treatment, either surgical or interventional, can be realized in reality. Several aspects, as for example the skill of the surgeon or cardiologist performing the treatment, intraprocedural complications or model uncertainties can and will affect the virtual and the real treatment. Thus, more data on how accurately a real intervention can be modeled *in silico* is required, as these uncertainties, such as in the angle or site of an anastomosis, might also have an effect on hemodynamic parameters such as HFD.

Finally, the assessment of viability of a given treatment strategy was mainly based on the experience of the heart team member in favor of the respective treatment, a general discussion, as well as some anatomical aspects as described in the methods section. A quantitative assessment of the procedural risk or chances of success, at least by independent rating by several members of the heart team, should be envisaged to allow to weigh the risks against the expected outcomes of different treatment strategies.

### Importance of sufficient image data quality and hybrid imaging approaches

According to Trusty et al., accurate prediction of the anatomy is one of the most important aspects to improve the accuracy of virtual prediction of HFD (21). In this study, accurate assessment of the entire anatomy of the TCPC using cardiovascular imaging was challenging in all three patients due to the presence of metallic artifacts which affected CMR as well as CT images. Furthermore, not only was a pronounced mismatch of HFD observed, but also the contrast agent administered during CT examination went predominantly to the pulmonary segments affected by PAVM, resulting in poor contrast in the unaffected sites. However, in general the CT images were superior for assessment of the patient-specific anatomy especially due to the high resolution and the ability to discern the metallic stents reasonably well. Especially the complex anastomosis sites of the Fontan conduit in patient #1 and of the azygos vein in patient #3 could not have been reconstructed from CMR images alone. Nonetheless, CMR images are mandatory for the approach to work, as patient-specific boundary conditions in all vessels are required to ensure reliable simulation results (20, 32). While most studies rely on CMR images for reconstruction of the patient-specific anatomy as well, these studies often focus on younger patients with less complex anatomical conditions, in whom implantation of different metallic artifacts has not yet been necessary (20). For complex cases as investigated in this study, additional acquisition of CT images might be necessary to assess the entire anatomy of the TCPC. While this procedure comes at the cost of exposure to ionizing radiation, the CT images were already valuable for surgical treatment-planning even without the virtual treatment-planning.

### Feedback from the cooperation between heart team members and the biomedical engineers

Even without the simulation of patient-specific hemodynamics before or after virtual intervention, the reconstruction of the patient-specific anatomy of the TCPC was already considered helpful for understanding the anatomical constraints and discussion of possible treatment strategies. Especially for the first two patients, where both image modalities were required to reconstruct the anatomy, the 3D model could be superimposed to either image data, enhancing their interpretability. For example, possible anastomosis sites and conduit orientations could be planned by replacing non-visible regions using the 3D model, which allowed to discriminate the location of the TCPC from surrounding cardiovascular structures or lung tissue.

Also, the pre-interventional simulation results were already seen as helpful for determination of treatment strategies, as they allowed investigation of the overall TCPC hemodynamics rather than investigation of isolated aspects of the flow by means of angiographic videos. These simulations also helped building trust in the general method, as they agreed well with the location of PAVM as well as angiographic findings.

### Limitations

This study is based on only three patients with a similar clinical background. While the anatomies and treatment histories of the patients differ widely, this sample size does not allow for general assumptions. However, the aim of this study was to demonstrate a proof-of-concept prediction of HFD in Fontan patients with PAVM and an extensive treatment history.

A quasi-steady numerical model was used, and all vessels were assumed to be rigid. The latter model assumption was chosen because the TCPC in this study featured different materials as vessel tissue, metallic stents, and vascular grafts, as well as scar tissue from previous surgeries, which would require combination of different constitutive models for which no data for parametrization was available. Previous studies suggest that the use of rigid walls is valid for time-averaged assessment of parameters such as HFD (35). However, for other aspects as for example the estimation of the energy or power loss in the TCPC, or the prediction of vascular remodeling at mid- to long-term this assumption might not be sufficient. Thus, identifying methods to parametrize patient-specific tissue properties, or at least assess whether tissue properties can be assumed based on general demographics in a sufficient manner, should be envisaged.

Virtual treatment strategies were only evaluated with regard to HFD optimization. No energetic aspects as pressure, or energy loss were investigated, which might result in conflicting recommendations (36). However, desaturation resulting from PAVM was the major clinical symptom in all patients, thus, optimization of HFD was considered the major aim for surgical or endovascular treatment.

Commonly, lumped parameter models are used to specify boundary conditions for parts of the TCPC. In this study, we only used 2D VEC CMR measurements to specify all boundary conditions. The good agreement between clinical examinations and the pre-interventional simulations support, that this choice of boundary conditions is also viable. However, this model does not allow to predict post-intervention changes in either flow rates or resistances. This becomes apparent by the HFD mismatch between the virtual and real treatment outcome in patient #3. While the anatomies of both simulations were very similar, the predicted HFD was only 5%, whereas the real HFD was 20%. However, if a treatment results in remodeling of PAVM, significant changes in the vascular resistance are to be expected. As this resistance is usually used for parametrization of lumped parameter models, those approaches might be suitable to assess hemodynamics assuming remodeling of PAVM by increasing vascular resistance accordingly.

## Conclusion

*In silico* assessment of the HFD before and after virtual treatment might allow to optimize patient-specific therapy planning in patients with pronounced hepatic flow mismatch and PAVM. However, this approach still requires intensive efforts with respect to validation and standardization before translation to clinical routine can be envisaged.

## Data availability statement

The raw data supporting the conclusions of this article will be made available by the authors, without undue reservation.

## Ethics statement

The studies involving human participants were reviewed and approved by Ethikkommission der Charité–Universitätsmedizin Berlin. Written informed consent to participate in this study was provided by the participants or their legal guardian/next of kin.

## Author contributions

MS, PY, SN, NS, and JB: data curation. MS, PY, and JB: formal analysis. MS, PY, PK, AS, FB, JP, YM, M-YC, SO, and JB: methodology. MS and JB: supervision. JB: visualization. MS, PK, and JB: writing – original draft. All authors contributed to the conceptualization, investigation, writing - review and editing, and approved the submitted version.
